# Intracapsular pressure and interleukin-1β cytokine in hips with acetabular dysplasia

**DOI:** 10.3109/17453671003717807

**Published:** 2010-04-06

**Authors:** Jun Xie, Masatoshi Naito, Akira Maeyama

**Affiliations:** Department of Orthopaedic Surgery, Fukuoka University School of MedicineJapan

## Abstract

Background and purpose Several studies have demonstrated an increased intracapsular pressure in several hip disorders such as septic arthritis, synovitis, and trauma. We therefore measured the intracapsular pressure in different positions in early dysplasic hips and its relation to the concentration of interleukin-1**β** (IL-1**β**), the volume of joint fluid, and the clinical and radiographic findings before a periacetabular osteotomy.

Methods 12 female patients (12 hips, mean age 35 (18–52)) with hip dysplasia were investigated. The intracapsular pressure was recorded and we investigated possible correlations with the Harris hip score, the Tönnis scale, radiographic findings, the volume of joint fluid, and the concentration of IL-1β.

Results An increased intracapsular pressure was noted, especially in flexion or extension with internal rotation. We found positive correlations between the intracapsular pressure and both the volume of joint fluid and the concentration of IL-1β.

Interpretation Increased intracapsular pressure varied with different positions, indicating the presence of synovitis resulting from early osteoarthritis in dysplastic hips. Positive correlations between the pressure and both the concentration of IL-1β and the volume of joint fluid suggest that the inflammatory cytokines produced by the synovial membrane as a consequence of mechanical instability of the hip joint may be of importance for the initiation and/or development of osteoarthritis in dysplastic hips.

## Introduction

Osteoarthrosis of the hip may be caused by mechanical abnormalities (Aronson 1986). In acetabular dysplasia, the reduced acetabular size and obliquity create shearing forces on the articular cartilage, and chronic overload of the cartilaginous and bony components of the anterior and anterolateral acetabular rim. If the chronic shear stress persists, the initially hypertrophied acetabular labrum will fail, and the labrum will be torn off the acetabular rim, sometimes with osseous fragment formation (Klaue et al. 1991, Mast et al. 1997). In addition to increased instability of the femoral head resulting from secondary labral lesions, the joint-sealing function, which is required for sufficient cartilage lubrication and distribution of joint pressures, is lost (Takechi et al. 1982, Ferguson et al. 2000). Once cartilage degradation has begun, the synovial membrane phagocytoses the breakdown products released into the synovial fluid such as glycosaminoglycans and type II collagen. Consequently, the membrane becomes hypertrophic and hyperplasic (Myers et al. 1990, Haraoui et al. 1991).

On the other hand, it is believed that cytokines and growth factors play an important role in the pathophysiology of osteoarthritis (OA). Cytokines appear to be first produced by the synovial membrane, and they diffuse into the cartilage through the synovial fluid. They subsequently activate the chondrocytes, which can in turn produce catabolic factors such as proteases and proinflammatory cytokines. During this vicious circle of events, interleukin-1β (IL-1β) is believed to be an important cause of cartilage destruction (Martel-Pelletier et al. 1999).

In the development of the OA process, changes in hydrostatic intracapsular pressure have been suggested as a predictor of cartilage degeneration (Goddard and Gosling 1988). In a normal hip joint, however, there is no increase in the intracapsular pressure within the normal range of rotation around the axis of the neck of the femur, only in the extreme rotatory positions (Wingstrand 1997). Several studies have demonstrated an increased intracapsular pressure in such hip disorders as arthrosis (Robertsson et al. 1995), septic arthritis, juvenile chronic arthritis (Hasegawa and Ito 1991), synovitis (Wingstrand et al. 1987), congenital dislocation of the hip (Wingstrand 1997), loosening of hip prosthesis (Robertsson et al. 1997), and trauma (Bonnaire et al. 1998). It is generally accepted that an increase in the intracapsular volume of fluid, due either to the bleeding following a trauma or to a septic/aseptic synovitis, contributes to an increased intracapsular pressure (Wingstrand et al. 1987, Bonnaire et al. 1998).

We investigated the intracapsular pressure in dysplasic hips and its relationship to clinical and radiographic evaluations, the volume of joint fluid, and the concentration of IL-1β in synovial fluid.

## Patients and methods

This study was approved by our institutional review board and informed consent from all patients was obtained preoperatively. 18 female patients with hip dysplasia were examined (18 hips); aspiration of synovial fluid failed in 6 cases and these hips were were excluded from the study, leaving 12 hips. The average age was 35 (18–52) years. None of the patients had any signs of avascular necrosis of the femoral head, had had previous hip infection, or had undergone surgical treatment. All patients had hip pain. The diagnosis of dysplasia was based on conventional radiography, in which the lateral center-edge angle (CE) was less than 20°. At the clinical evaluation, the Harris hip score was determined (Harris 1986). The CE angle, the Sharp angle, and the acetabular-roof angle were measured on anteroposterior (AP) radiographs. The radiographic severity of osteoarthritis was scored using the Tönnis scale (Tönnis and Trousdale 1999). Measurement of intracapsular pressure was performed with the patient in supine position after general anesthesia, before a curved periacetabular osteotomy. An intracapsular pressure testing apparatus (Millar Instruments, Houston, TX), which is composed of a pressure transducer at the end of a catheter connected to a digital manometer, and an 18-gauge side-port needle, was used to determine the intracapsular pressure (Boody and Wongworawat 2005). The side-port needle inserted with the transducer was injected into the hip joint superiorly to the greater trochanter and the location of the needle was confirmed under a C-arm image intensifier. The pressure data were recorded during a 5–10 second interval, starting with the hip in a spontaneous position (slight flexion and abduction), followed by 45° abduction, 30° adduction, 90° flexion, 90° flexion with 45° of internal rotation (FIR) and external rotation (FER), and finally in extension with 30° of internal rotation (EIR) and external rotation (EER) (radiographically controlled).

### Aspiration of joint fluid and measurement of interleukin 1*β* (IL-1*β*)

In a pilot study, it was difficult to aspirate the synovial fluid in some patients because there was no or little effusion. Thus, any intracapsular effusion in the hip joint was first detected preoperatively by sonography, and then the synovial fluid was aspirated and subjected to determination of IL-1β concentration as described below. 20 mL of 0.9% saline solution was injected into the hip joint and the hip was flexed several times to enable a homogeneous dilution of synovial fluid. 2–10 mL of synovial fluid was aspirated using an 18-gauge needle and kept at –80 degrees. When all samples were collected, the concentration of IL-1β was examined using a commercially available kit according to the manufacturer's instructions (Cayman Chemical Company, Ann Arbor, MI). Briefly, 100 μL of sample was added to each well of a 96-well plate coated with a monoclonal antibody specific for IL-1β. 100 μL of Fab' conjugate, an acetylcholinesterase selectively bound to a different epitope on the IL-1β molecule, was also added to the well. After incubation overnight at 4°C, the wells were emptied and rinsed 5 times with a wash buffer, and 20 mL of Elleman's reagent was then added to each well. The plate was placed in an orbital shaker in the dark for 30 min. The concentration of IL-1β was then determined spectrophotometrically at 412 nm.

### Statistics

Possible correlations between intracapsular pressure and clinical Harris score, radiographic data (CE angle, acetabular-roof angle, Sharp angle), degree of osteoarthritis, volume of aspirate, and the concentration of IL-1β were examined. The data were subjected to an analysis of variance with the Scheffe post hoc test and the Pearson correlation coefficient was used for statistical analysis using Statview version 5.0 (SAS Institute, Cary, NC), with the significance level set at 0.05.

## Results

**Figure 1.**
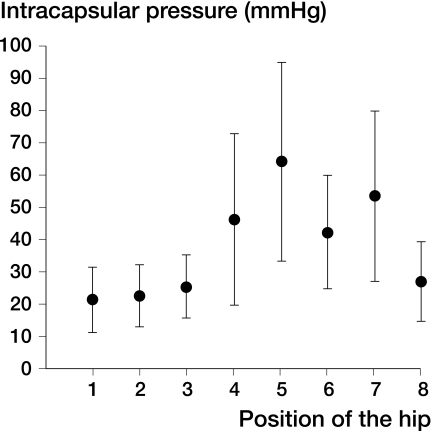
Intracapsular pressure in different positions of the hip. (1) spontaneous; (2) 45° abduction; (3) 30° adduction; (4) 90° flexion; (5) flexion with internal rotation (FIR); (6) flexion with external rotation (FER); (7) extension with internal rotation (EIR); (8) extension with external rotation (EER).

The mean Harris hip score was 76 (53–88). The average values for CE angle, Sharp angle, and acetabular-roof angle were 12 (6–18), 46 (43–50), and 18 (5–25), respectively. The severity of OA scored on the Tönnis scale was 1.6 (1–3). The highest pressure was noted in flexion (75 (SD 39) mmHg) and extension (59 (SD 24) mmHg) with internal rotation (Table and Figure 1). Pearson's correlation coefficient analysis showed no correlation between the intracapsular pressure at any position and the clinical Harris hip score, nor with radiographic findings including the Tönnis scale.

**Table:** Intracapsular pressure in various positions, clinical and radiographic findings, and concentration of IL-1β

Case:	1	2	3	4	5	6	7	8	9	10	11	12
Age	36	26	28	18	33	27	52	45	40	42	42	31
Spontaneous	14	31	7	21	26	13	4	27	27	30	38	21
Abduction	19	29	32	19	19	7	26	24	45	13	16	23
Adduction	19	23	27	16	32	18	28	21	46	41	20	16
90° flexion	21	30	37	95	25	21	27	60	30	83	47	79
FIR **^a^**	25	45	49	52	67	30	78	90	69	142	59	66
FER **^b^**	26	21	21	46	42	36	84	46	40	61	39	47
EIR **^c^**	29	37	39	54	19	27	75	67	67	106	80	40
EER **^d^**	13	30	17	35	28	11	21	37	21	56	35	20
Harris score	76	84	53	55	86	81	84	78	72	70	85	88
Tönnis scale	1	1	1	1	2	1	1	3	2	2	3	1
CE angle	15	10	15	15	10	6	10	7	7	15	18	12
Roof angle	16	18	9	5	20	20	22	19	18	25	21	20
Sharp angle	45	50	46	43	40	49	50	46	45	44	44	45
Volume of fluid	3	9	4	3	10	6	2	8	6	10	9	4
IL-1ß (pg/mL)	24	66	53	42	57	60	52	59	56	64	74	55
**^a^** FIR, internal rotation in flexion;												
**^b^** FER, external rotation in flexion;												
**^c^** EIR, internal rotation in extension;												
**^d^** EER, external rotation in extension												

**Figure 2.**
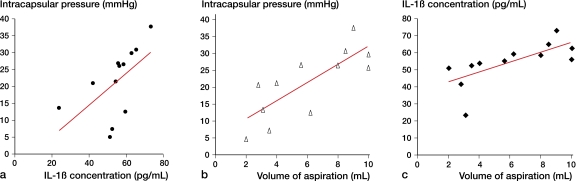
Positive correlations were found between intracapsular pressure and both the concentration of IL-1β in synovial fluid (a) and the volume of joint fluid (b). There was also a positive correlation between the concentration of IL-1β and the volume of joint fluid (c).

On average, 6 (2–10) mL of intracapsular fluid was aspirated. The mean concentration of IL-1β in synovial fluid was 55 (SD 12), ranging from 24 to 74 pg/mL. A positive correlation was observed between the concentration of IL-1β and the intracapsular pressure in the spontaneous position (r = 0.60, p < 0.05) (Figure 2a), and also between the volume of aspiration and both the intracapsular pressure (r = 0.78, p < 0.05) (Figure 2b) and the concentration of IL-1β (r = 0.69, p < 0.05) (Figure 2c).

## Discussion

The intracapsular pressure varied with different positions, and the highest pressures were noted in flexion and extension with internal rotation when the joint capsule is tightened, leading to narrowing of the capsular cavity. This is consistent with the results of previous studies (Hasegawa and Ito 1991, Robertsson et al. 1995).

We did not find any statistically significant correlations between intracapsular pressure and the clinical or radiographic findings. The Harris hip score is of limited value when evaluating clinical symptoms, especially at an early stage of hip dysplasia (average Tönnis scale: 1.6). Another explanation for the lack of correlation may have been the small number of hips. In theory, the severity of OA, as evaluated by radiologically visible joint degeneration, is associated with increased intracapsular pressure. That is, more synovitis and synovial fluid and higher tension in the joint capsule in less severe case of OA results in drainage problems for joint fluid through subsynovial veins, leading to higher intracapsular pressure (Arnoldi 1972). As the OA progresses, a dry joint develops with less intraarticular fluid leading to reduced intracapsular pressure. This is the usual preoperative finding in the late stages of OA (Tarasevicius et al. 2007). Thus, it would be of interest to correlate the radiological degeneration with the amount of joint effusion, which may be evaluated by sonography or magnetic resonance imaging (MRI).

Inflammatory cytokines are believed to play a pivotal role in the initiation and development of the OA process; among them, IL-1β appears to be prominent (Jacques et al. 2006). We found a higher concentration of IL-1β in dysplastic hips. This is in agreement with an animal study that reported an increase in markers of osteoarthritis in dogs with acetabular dysplasia (including IL-1β, IL-6, tumor necrosis factor (TNF)-alpha, and matrix metalloproteinase (MMP)-3) in comparison to normal dogs (Fujita et al. 2005). In addition, we found a moderate, positive correlation between the intracapsular pressure and both the concentration of IL-1β and the volume of joint fluid. We assume that IL-1β is produced by synovial membrane or macrophages within the joint as a consequence of mechanical irritation, due to joint instability or labrum rupture, or as a consequence of synovitis including effusion. This effusion has a high concentration of IL-1β with an activating effect on chondrocytes, which could in turn produce catabolic factors such as proteases and inflammatory cytokines, resulting in cartilage destruction. The existing damage to cartilage may also contribute to this vicious circle. However, one cannot assume that the correlation between the intercapsular pressure and the concentration of IL-1β on the one hand and between the inflammatory response and intracapsular pressure on the other are pairs involving any kind of rigid causality. The truth is more complex.

We conclude that intracapsular pressure is elevated in hip dysplasia, that it varies with the position of the hip, and that there is a correlation between an increase in IL-1β cytokine and both an increase in the volume of joint fluid and a change in intracapsular pressure. However, the value of our study is limited by the lack of information on normal hips from healthy people.
